# Maternal Serum Angiogenic Profile and Its Correlations with Ultrasound Parameters and Perinatal Results in Normotensive and Preeclamptic Pregnancies Complicated by Fetal Growth Restriction

**DOI:** 10.3390/jcm12134281

**Published:** 2023-06-26

**Authors:** Weronika Dymara-Konopka, Marzena Laskowska, Ewelina Grywalska, Anna Hymos, Bożena Leszczyńska-Gorzelak

**Affiliations:** 1 Department of Obstetrics and Perinatology, Medical University of Lublin, 8 Jaczewskiego Street, 20-095 Lublin, Poland; bozena.leszczynska-gorzelak@umlub.pl; 2 Department of Experimental Immunology, Medical University of Lublin, 4a Chodźki Street, 20-093 Lublin, Poland

**Keywords:** fetal growth restriction (FGR), preeclampsia, placental growth factor (PlGF), soluble endoglin (sEng), soluble fms-like tyrosine kinase-1 (sFlt-1), sFlt-1/PlGF ratio, Doppler ultrasound, risk prediction

## Abstract

FGR is a complication of pregnancy in which the fetus does not reach its programmed growth potential due to placental reasons and it is the single largest risk factor of stillbirth. Babies with FGR are at increased risk of mortality and morbidity not only in the perinatal period, but also in later life. FGR presents a huge challenge for obstetricians in terms of its detection and further monitoring of pregnancy. The ultrasound is the gold standard here; apart from assessing fetal weight, it is used to measure Doppler flows in maternal and fetal circulation. It seems that additional tests, like biochemical angiogenic factors measurement would be helpful in diagnosing FGR, identifying fetuses at risk and adjusting the surveillance model. The study aimed to assess the potential relationship between the concentration of sEng, sFlt-1, PlGF, and the sFlt-1/PlGF ratio in maternal serum at delivery and maternal and fetal Doppler flow measurements as well as perinatal outcomes in pregnancies complicated by FGR with and without PE, isolated PE cases and normal pregnancies. The use of angiogenic markers is promising not only in PE but also in FGR. Numerous correlations between ultrasound and Doppler studies, perinatal outcomes and disordered angiogenesis marker levels in maternal serum suggest that biochemical parameters have a great potential to be used as a complementary method to diagnose and monitor pregnancies with FGR. The, PlGF in particular, could play an outstanding role in this regard.

## 1. Introduction

Fetal growth restriction (FGR) is a complication of pregnancy in which the fetus does not reach its programmed biological growth potential due to placental reasons [1,2,3]. FGR is a huge challenge for obstetricians in terms of its diagnosis and further monitoring of pregnancy. Detection of FGR is based on the identification of a fetus that is smaller than expected for gestational age, through either physical examination (symphysis–fundal height, SFH) or ultrasound, which is a cornerstone of medical examination [3]. Standard fetal biometry includes assessment of head circumference (HC), biparietal diameter, abdominal circumference (AC), and femur length (FL). Fetal weight is estimated based on various combinations of the four biometric indices described above, with the Hadlock equation based on three indices (HC, AC, FL) providing the greatest accuracy, according to a recent systematic analysis [4,5]. FGR can be defined as an estimated fetal weight—EFW/AC < 3rd percentile or absent end-diastolic velocity in the umbilical artery (UA AEDV) or EFW < 10th percentile in combination with at least one of the following Doppler abnormalities: umbilical artery (UA) pulsatility index (PI) > 95th percentile, cerebroplacental ratio (CPR) < 5th percentile, and/or a mean uterine artery (mUtA) PI > 95th percentile, depending on gestational age. The complexity of diagnostic criteria is representative of how complicated the syndrome is.

FGR and PE are both representative of the group of great obstetrical syndromes [6,7]. In many cases, they occur together, overlapping clinically, especially in early-onset forms [8,9,10]. In either case, the placenta is the key problem, and its abnormal development and malfunction play a major role in the pathogenesis of both preeclampsia (PE) and fetal growth restriction (FGR) [11,12]. Placental function is a critical regulator of fetal growth and development, as well as a mediator of fetal programming; thus, both FGR and PE expose children to long-term health problems [13,14]. Babies with FGR are at increased risk of mortality and morbidity not only in the perinatal period but also in later life [9,15,16]. Mothers of babies with impaired growth are at increased risk of developing PE [17,18]. The appearance of fetal growth restriction in the course of PE implies an upgrade in severity of the course of FGR [8,9,19]. Substances regulating angiogenesis, secreted by the placenta, are interpreted as markers of its dysfunction in these two pathological conditions, and in recent years, they have been of great interest both among researchers and obstetricians, who have proposed an increasingly wider practical application for them. In particular, the sFlt-1/PlGF ratio is being implemented for prediction, diagnosis, and prognosis of disease evolution in algorithm-based recommendations for PE [20,21].

However, it is now the subject of research to determine whether the significance of angiogenic factors in PE may be extrapolated to FGR as a part of the clinical picture of placental ischemic disease [22,23,24,25,26,27]. Much data suggest that combining ultrasound parameters (fetal biometry, feto-maternal Doppler studies) and angiogenic marker levels as well as the sFlt-1/PlGF ratio appears to be useful as a supplementary criterion not only for the detection of FGR but also for the prediction of the time-to-delivery interval and associated adverse outcomes in isolated FGR cases and is increasing [28,29,30,31,32].

The study aimed to assess the potential relationship between the concentration of sEng, sFlt-1, PlGF, and the sFlt-1/PlGF ratio in maternal serum with the necessity of delivery because of the risk to the mother and fetus and the correlation with angiogenic substances, with fetal Doppler flow measurements as well as perinatal outcomes in pregnancies complicated by FGR with and without PE, isolated PE cases and normal pregnancies.

The study also aimed to assess the possibility of using the tested vasoactive substances in clinical practice in anticipation and prevention of risk to the mother and/or especially to the fetus and qualifying labor in order to avoid unfavorable pregnancy outcomes (for example, fetal demise).

## 2. Materials and Methods

### 2.1. Patients

A prospective cross-sectional case-control study was conducted on patients aged 20–41 years, between 24 and 41 weeks of gestation. A total of 77 pregnant women out of the 88 initially involved fulfilled the criteria for their inclusion in the study. Eligible cases were live singleton pregnancies with a diagnosis of fetal growth restriction (FGR) with or without concurrent PE as well as preeclamptic patients without FGR hospitalized in a Polish tertiary referral hospital. Seventy-seven women were included: 36 had pregnancies complicated by FGR, of whom 14 were isolated FGR cases (the iFGR group) and 22 were FGR with concurrent preeclampsia (FGR + PE group); 21 patients suffered from isolated preeclampsia (iPE group); and the control group consisted of 20 healthy pregnant women without any complications or disorders and with appropriate gestational age intrauterine fetal growth. We used the most recent criteria established by the experts: FGR was diagnosed according to the Delphi consensus-based definition for placenta-mediated FGR published by Gordijn et al. in 2016, recognized recently in 2021 by the International Federation of Gynecology and Obstetrics (FIGO) initiative on fetal growth, which uses a combination of measures of fetal size percentile and Doppler abnormalities for early and late FGR (Table 1). Preeclampsia (PE) was defined according to the criteria applied in 2018 by the International Society for the Study of Hypertension in Pregnancy (ISSHP) Group: the new onset of hypertension (BP ≥ 140 mm Hg systolic or ≥90 mm Hg diastolic) on two or more consecutive occasions accompanied by new-onset proteinuria (>0.3 g/24-h in 24-h urine collection) or, in the absence of proteinuria, another maternal organ or uteroplacental dysfunction (Table 2).

Healthy normal pregnancies were recruited in our outpatient department to gestationally match the pathologic cases. All patients from the control group were pairwise matched with FGR/PE patients by gestational age at the moment of blood sapling and Doppler ultrasound examination. Inclusion criteria were noncomplicated singleton pregnancy with absence of labor at the time of venipuncture. Postdelivery, the estimated fetal weight/birth weight was compared to local birth weight charts to exclude false prenatal diagnosis and to ensure that the control group included only women with delivery of a term (>37 weeks) infant whose birth weight was between the 10th and 90th percentiles for gestational age and there were no medical, obstetrical or surgical complications during the entire gestation.

### 2.2. Methods

Blood samples were collected within 48 h before delivery due to fetal or maternal clinical aggravation where the differences in terms of angiogenic imbalance were expected to be maximized. The levels of placental growth factor (PlGF), soluble endoglin (sEng) and soluble fms-like tyrosine kinase-1 (sFlt-1) in maternal serum were measured by the corresponding sandwich enzyme immunoassay technique kits (R&D Systems Europe Ltd., Abingdon, UK) according to the manufacturer’s instructions.

Fetal measurements and Doppler studies were performed at the Clinical Department of Obstetrics and Perinatology using a curvilinear transabdominal probe and a Voluson E10 device (GE Medical Systems). The ultrasound was carried out by senior obstetricians upon routine conditions and guidelines. Biometry was performed by measuring the abdominal circumference (AC), the biparietal diameter (BPD), head circumference (HC) and the femur length (FL). The fetal weight and the weight percentile were calculated using the Hadlock curves [34]. The following Doppler parameters were measured: PI (pulsatility index) and RI (resistance index) of the uterine arteries (UtA), PI and RI of the umbilical artery (UA), PI and RI of the middle cerebral artery (MCA) and the cerebroplacental ratio (CPR) as the ratio between MCA PI and UA PI. Calculations were performed according to up-to-date reference ranges [35,36].

Fetal measurements and Doppler studies in investigated groups were performed within 48 before delivery, as in many cases they were part of the protocol of close fetal surveillance and constituted indications for delivery. Only the last results before the delivery were included for analysis and their correlation with angiogenic substances was studied as the aim of the study.

Blood samples from the control group were collected from healthy patients pairwise matched with FGR/PE patients by gestational age. Fetal measurements and Doppler studies in the control subjects were performed in these healthy pregnant patients pairwise matched with FGR/PE patients by gestational age.

### 2.3. Statistical Analysis

All statistical analyses were conducted using Statistica 13.1 by StatSoft. To analyze correlations between angiogenic growth factor levels and Doppler parameters, the Spearman’s correlation coefficient was calculated. A two-sided *p* < 0.05 was considered statistically significant. The analysis was conducted with the Kruskal-Wallis test with Bonferroni’s adjustment and analysis of variance ANOVA with post hoc RIR Tukey test when possible.

The study protocol was approved by the Medical University of Lublin Ethics Committee (KE-0254/258/2016). Written consent was obtained from all participants in the study.

## 3. Results

Patients demographic data, clinical characteristics and biochemical test results are presented in Table 3.

There were no statistically significant differences with regard to gravidity and parity, maternal age, weight, or height in patient profiles between groups. In the FGR complicated by simultaneous PE (FGR + PE), the mean gestational age at delivery was lower than in the isolated FGR/isolated PE groups (group iFGR and iPE group) (median value 32 vs. 35 weeks, *p* < 0.05). The systolic blood pressure (SBP) and mean arterial blood pressure (MAP) values were significantly higher in all investigated subgroups in comparison with the control group (*p* < 0.05), and diastolic blood pressure (BDP) was higher than controls in the two PE groups (FGR + PE and iPE; *p* < 0.05). Aspartate and alanine transferases (AST and ALT), uric acid (UAc) and urea values were higher in preeclamptic patients in both groups (FGR + PE and iPE) than in healthy controls (*p* < 0.05).

Sonographic parameters including Doppler flow evaluation as well as perinatal outcomes are presented in Table 4.

Of the patients with pregnancies complicated by FGR, 83.33% (30/36) had abnormal Doppler study results (UtA or UA PI above the 95th percentile or MCA PI or CPR under the fifth percentile), and the remaining 16.67% (6/36) of patients with pregnancies complicated by FGR had exclusively EFW (estimated fetal weight) under the third percentile. The Doppler study analysis revealed a statistically higher uterine artery mean pulsatility index (UtA PI) in both FGR groups (iFGR, FGR + PE) and a higher umbilical artery pulsatility index (UA PI) in all investigated subgroups compared to controls. Serum concentration of PlGF was lower, but sEng and the sFlt-1/PlGF ratio were higher compared to the control group in all patients with pregnancies complicated by fetal growth restriction (iFGR, FGR + PE groups) and in preeclamptic pregnancies without FGR (iPE group). Serum sFlt-1 concentrations significantly higher and different from the control group were found only in preeclamptic patients (iPE and FGR + PE groups).

Serum levels of investigated angiogenic markers, PlGF, sEng, sFlt-1 and sFlt-1/PlGF ratios, are presented in Table 5.

**Figure 1:**
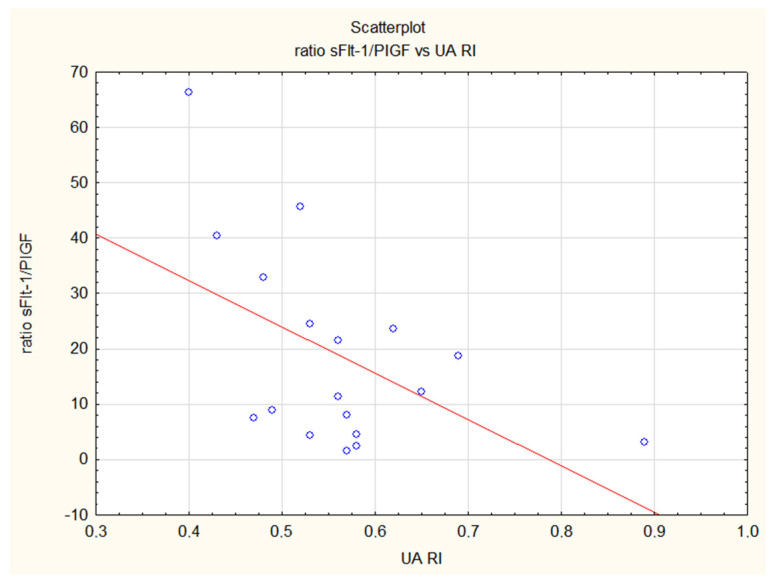
Inverse correlation of the sFlt-1/PlGF ratio and the resistance index in the umbilical artery (UA RI) in the control group. The ratio is increasing with advancing gestational age and resistance in umbilical artery in physiological pregnancies is decreasing (R = −0.49, *p* = 0.04).

**Figure 2:**
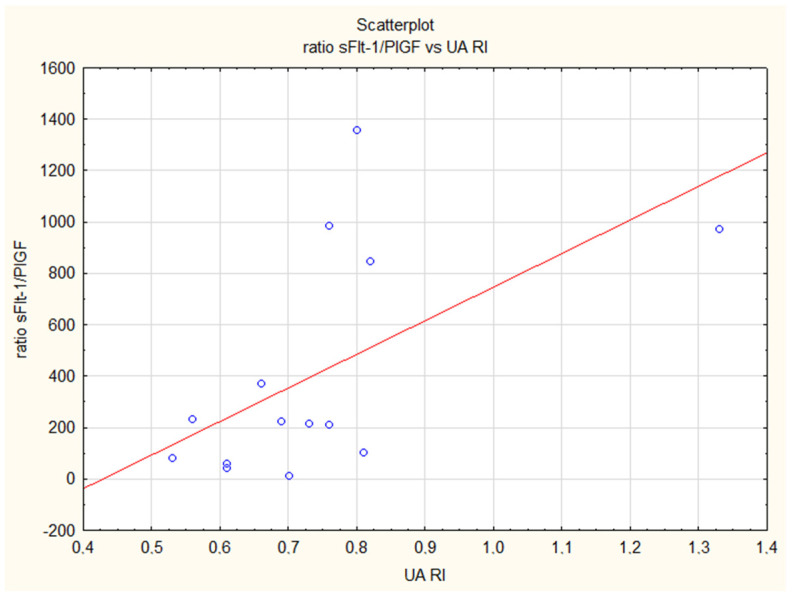
Positive correlation of the sFlt-1/PlGF ratio and the resistance index in the umbilical artery (UA RI) in the iFGR group (R = 0.57, *p* = 0.05).

Regarding sonographic measurements, in the control group we observed a positive correlation between EFW, AC and levels of sFlt-1 as well as the sFlt-1/PlGF ratio. There was a positive correlation between the level of PlGF and the following Doppler flow measurements: UA PI, UA RI and MCA PI. There was a negative correlation between UA PI and RI and MCA PI with levels of sEng. The sFlt-1/PlGF ratio was correlated negatively with UA RI (Figure 1) and MCA PI in healthy pregnancies. In all preeclamptic women (the FGR + PE and iPE groups of patients), we noted a negative correlation between PlGF and UtA PI, UA PI and UA RI but a positive correlation between this angiogenic factor and CPR. In PE patients, PlGF was additionally positively correlated with MCA PI. In the isolated FGR group, there was a positive correlation between the sFlt-1/PlGF ratio and UtA PI and UA RI (Figure 2) and a negative correlation between concentrations of sFlt-1 and MCA PI and CPR (cerebroplacental ratio). Table 6.

**Figure 3:**
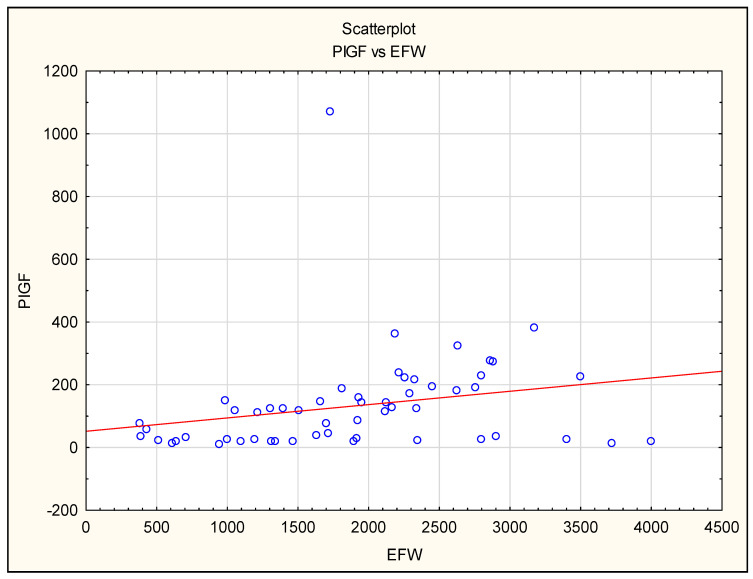
The correlation between placental growth factor (PlGF) maternal serum concentration before delivery and estimated fetal weight (EFW) in all pregnancies complicated by FGR and/or PE.

We noted a positive correlation between PlGF levels and EFW in all preeclamptic patients, in the FGR + PE group and in the isolated FGR group (Figure 3).

The soluble form of endoglin presented an inverse correlation with EFW in PE (all preeclamptic patients including FGR + PE and iPE groups of pregnant women) and iFGR groups but a positive correlation with UA PI in all preeclamptic patients (FGR + PE and iPE groups).

**Figure 4:**
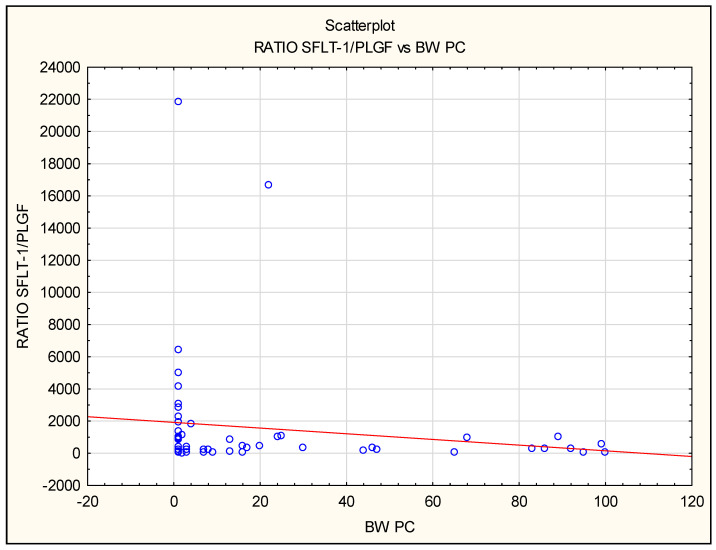
The correlation between sFlt-1/PlGF ratio before delivery and birth weight expressed in centiles for gestational age in all pregnancies complicated by FGR and/or PE. The negative correlation was found using Spearman’s rank correlation in the isolated FGR group (iFGR: R = −0.62, *p* = 0.02) and in all preeclamptic patients (iPE and FGR + PE: R = −0.9, *p* = 0.009).

Numerous associations between levels of biomarkers in maternal serum and perinatal outcomes were observed in FGR cases, both isolated FGR as well as FGR additionally complicated by PE: PlGF correlated positively, whereas the sFlt-1/PlGF ratio and sEng correlated negatively with birth weight (BW) of infants (Figure 4). A positive correlation between PlGF and the Apgar score at 1st and 5th minute was noted in the iFGR group and all PE patients together (FGR + PE and iPE groups) Table 7.

We showed a positive correlation of PlGF concentrations with INR results in the entire study population of pregnant women. Such a correlation was also observed in the iFGR group. A negative correlation between the sFlt-1/PlGF index and INR was observed in both PE groups and in the iFGR group. An inverse correlation of PlGF concentrations with systolic blood pressure (SBP) and MAP values was found in the entire study population of pregnant women, and this correlation was confirmed in a smaller group of patients with iFGR. Table 8.

The positive correlation was found using Spearman’s rank correlation in the iFGR group (R = 0.6, *p* = 0.02), the FGR and PE groups (R = 0.5 *p* = 0.02) and in all preeclamptic patients (iPE and FGR + PE: R = 0.43, *p* = 0.005).

## 4. Discussion

The detection of FGR is still unsatisfactory, even in well-developed countries. The ultrasound is of primary usefulness here, which, apart from assessing fetal weight, is used to measure blood flow in the uterine, umbilical and middle cerebral arteries at diagnosis and monitoring of the fetus, as their abnormal pattern is known to change in a characteristic way [3,37,38]. The management of FGR complicated by concurrent PE should be combined with monitoring of preeclamptic women, which also takes into consideration the maternal state and the possibility of rapid aggravation of fetal well-being in this condition [39]. Maternal symptoms, such as blood pressure and biochemical markers, reflect the severity of the disease for the mother, but their use for estimating fetal well-being is limited. The severity of FGR can be determined with Doppler flow measurements, which are an integral part of the diagnostic process and fetal surveillance.

In our study, 83.33% of patients with FGR had abnormal Doppler study results (UtA/UA above the 95th percentile or MCA/CPR under the fifth percentile). The groups with FGR had a median UtA PI value as high as 1.6, which corresponded to the 100th percentile for gestational age at the moment of delivery. In the isolated PE group, the median UtA PI was slightly lower at 1.0, which corresponded to the 91st percentile for gestational age.

However, the results of Doppler studies may be somewhat delayed in relation to the onset of insufficiency, as some studies have shown quite severe ischemic placental lesions despite the diagnosis of normal UA flows [40]. It has been suggested that the prediction of FGR based on routine third-trimester ultrasound can be improved by integrating EFW with additional biomarkers, like angiogenic factors [3].

The most numerous correlations in all studied groups of the present study apply to the placental growth factor. PlGF has been positively correlated with EFW in all preeclamptic patients, as well as in pregnancies complicated by fetal growth restriction: FGR + PE and iFGR groups. The higher the PlGF value, the better the fetal growth, despite the diagnosis of PE and/or FGR.

In all studied preeclamptic patients (with and without FGR), the PlGF level was inversely correlated with the value of the pulsatility index in uterine arteries (UtA PI) shortly before the necessity of emergency delivery. The lower the level of PlGF, the greater the disturbances and the greater the flow resistance in UtA. We did not find such a correlation in the iFGR group, although there was a positive correlation between the sFlt-1/PlGF ratio and UtA PI in this group. Kwiatkowski et al. also found such a correlation between UtA PI and the sFlt-1/PlGF ratio in the population of patients with small-gestational-age fetuses (SGA) [41].

Uterine artery blood flow reflects physiological changes in feto–maternal circulation during pregnancy. Doppler flow measurements provide important information on the process of conversion of the spiral arteries into uteroplacental arteries. In physiological pregnancies, the UtA diameter is increasing and blood flow is increasing and characterized by continuous, significant decline in resistance indices including the mean UtA PI with advancing gestation [35,42]. Abnormal placentation, which may cause early FGR and PE, results in an increment of the resistance index (RI) and pulsatility index (PI) of UtA [35,43,44,45].

The study of UtA flows has been used in the screening and diagnosis of forms of placental insufficiency, such as PE and FGR; hence, the above-described relationships between UtA and the concentration of angiogenic factors prove their potential role as additional markers indicating its malfunction. They could be used as an auxiliary diagnostic and prognostic tool not only in preeclampsia itself but also in the related complication of pregnancy, which is FGR.

Verlohren et al. described an unequivocal negative correlation of the uterine artery resistance index (UtA RI) with birth weight, which is evident in both early-onset and late-onset preeclampsia [46]. It was also observed that an abnormal Doppler flow in uterine arteries also confers a high risk of intrapartum fetal distress, emergency Cesarean section and admission to the neonatal intensive care unit (NICU) [47,48].

Similarly, a negative relationship between maternal levels of PlGF and the pulsatility index in UtA (UtA PI) as well as in the umbilical artery (UA PI) in pregnancies complicated by PE and/or FGR just before delivery was found by Schlembach et al., indicating the combination of these two parameters—Doppler examination and PlGF concentration—as a future screening tool for these pregnancy complications [49]. Kienast et al. proved that disturbed blood flows in uterine arteries and low PlGF values allow the identification of fetuses with growth restriction [50].

In light of the established positive relationship between PlGF and EFW in our research in all groups with pregnancies complicated by preeclampsia and/or fetal growth restriction (the PE, iFGR and FGR + PE groups of studied pregnant women), we support the hypothesis that PlGF might be a helpful tool in FGR pregnancy identification.

Gaccioli et al. noted that low PlGF in fetuses with suspected SGA identifies women at increased risk of adverse perinatal outcomes [29].

In our study, the highest values of sFlt-1 were observed in patients with pregnancies complicated by FGR in the course of preeclampsia (the FGR + PE group), where abnormalities in Doppler examinations in UtA and UA were also more pronounced than in the group with isolated PE (median PI for UtA and UA in the PE + FGR group corresponded to the 100th percentile for gestational age and 84th percentile, respectively, compared to iPE—91st percentile and 73rd percentile or gestational age, respectively). Abnormal, high concentrations of sFlt-1 lead to a decrease in PlGF levels in pregnancies complicated by ischemic placental syndrome [22,23,51,52]. In addition to an increased release of sFlt-1, which binds free PlGF, reducing its bioavailability, uteroplacental ischemia may downregulate expression and production of PlGF [49].

Chaiworapongsa et al. proved that among pregnancies complicated by FGR, the level of sFlt-1 in maternal serum was increased only in cases with abnormal uterine artery blood flows and the magnitude of the sFlt-1 increase is related to Doppler abnormalities in the maternal and fetal circulation, which is in line with our results [53]. In their study, the highest sFlt-1 concentrations reached patients diagnosed with PE or with SGA babies with abnormal uterine and umbilical arteries blood flows, fulfilling modern criteria of placental FGR [53]. In our study, we did not find any direct correlation between sFlt-1 and ultrasound parameters in the FGR + PE group, which was the group with the highest sFlt-1 concentration. However, low PlGF values—comparable with those in groups with isolated PE/isolated FGR cases—had numerous correlations with ultrasound measurements in this group, as described above.

As observed in our study, the relationship between low PlGF in preeclamptic pregnancies complicated by FGR with high resistance parameters in UtA indicates a greater contribution of abnormal placentation in early forms of PE and FGR, which is also indicated by other literature sources [8,9,19]. It provides evidence of a certain accumulation of angiogenic imbalance in these forms of FGR and PE, resulting in the strongest alterations in Doppler ultrasound examinations in the group of patients with coexisting FGR and PE, which was also characterized by a lower gestational age at delivery than in the group with isolated PE or FGR (median gestational age was 32 vs. 35 weeks, *p* < 0.05).

We also found in preeclamptic pregnancies complicated by fetal growth restriction that PlGF was negatively correlated with Umbilical Artery (UA) PI and UA RI. UA Doppler is the only measure that provides both diagnostic (alone or in combination with MCA—CPR ratio) and prognostic (as the progression of UA Doppler patterns to absent or reverse end-diastolic flow correlates with the risks of injury or death) information for the management of FGR [3,39].

The umbilical artery Doppler waveform can be quantified using the pulsatility index, or by visual classification of end-diastolic velocity as absent (AEDV) or reversed (REDV). With increasing degrees of placental blood flow resistance, an abnormal umbilical artery waveform is defined as either having an elevated pulsatility index, AEDV or REDV.

In assessing fetal well-being in FGR cases, UA flow is one of the key elements of ultrasound scan. There is compelling evidence that using UA Doppler in high-risk pregnancies (most of them SGA fetuses) improves perinatal outcomes, with a 29% reduction (2–48%) in perinatal deaths [54]. There is an association between reversed end-diastolic flow in the UA and adverse perinatal outcomes [37]. Absent or reversed end-diastolic velocities (AEDV/REDV), the end of the spectrum of the abnormalities of the UA Doppler, have been reported to be present on average 1 week before the acute deterioration [55]. Up to 40% of fetuses with acidosis show this umbilical flow pattern. Reversal end-diastolic flow (REDF) justifies delivery after 30 weeks, since the risk of stillbirth for the fetus outweighs the risk of prematurity, whereas absent end-diastolic flow (AEDF) is an indication for delivery at 32 weeks [3,56,57]. In the aforementioned first situation, there is evidence of damage to almost 80% of chorionic villi and to 60% of villi in the second [58].

We established that positive correlations of the sFlt-1/PlGF ratio and maternal sEng levels with UA PI and UA RI in isolated FGR and PE patients as well as a negative correlation of PlGF serum concentration with these indices in the FGR + PE group at 48 h before delivery reflect the severity of placental pathology expressed by angiogenic substance alterations in these complicated clinical situations, which are indicative of imminent delivery. These correlations allow us to presume that by combining different surveillance tools, like ultrasound UA Doppler flows and biochemical angiogenic factors levels, better identification of high-risk pregnancies and fetuses at risk may be possible.

In our study in the iFGR group, we found a negative correlation between the sFlt-1 level and fetal MCA PI and CPR expressed in percentiles for gestational age. The higher the sFlt-1 concentration in the maternal serum was, the higher the end-diastolic velocity, the lower the MCA resistance and the lower the pulsatility index (PI). In the iFGR group, we also found a negative correlation between the sFlt-1 concentration and birth weight expressed in percentiles for gestational age (BW pc).

In the currently ongoing RATIO 37 study, researchers use Doppler measurements to decide whether to induce labor at 37 weeks of gestation in the case of estimated fetal weight <10th percentile and abnormal CPR, i.e., <5th percentile [45]. The correlation of the sFlt-1 level with flow measurements in the MCA and CPR as well as BW in the iFGR group, which we observed, indicates the potential role of this substance as an additional sought-after tool in the care of pregnancy with FGR.

Conde-Agudelo et al. found that the CPR index itself has moderate accuracy in predicting low birth weight in pregnancies suspected of FGR, but it allows predicting perinatal death in fetuses with growth disorders [59]. Due to the latter, the most serious and at the same time often unexpected—especially in late forms—complication of FGR, new methods are still being sought to identify fetuses at high risk of intrauterine death. High sFlt-1 in iFGR correlates with low birth weight and predicts fetal risk and features of fetal circulation centralization, which may constitute an indication for increased fetal surveillance involving adjusting the frequency of USG and cardiotocography (CTG) examinations, ultimately contributing to improved perinatal outcomes.

In our study results, sFlt-1 was elevated in the iFGR group compared to the control group, but the differences turned out to be statistically insignificant. However, sFlt-1 as well as sEng levels, which are positively coupled in all investigated subgroups, correlate negatively with birth weight in the iFGR group, and for sEng, this relationship is also present in the PE groups. Similarly, the sFlt-1/PlGF ratio shows a negative correlation with BW in both the iFGR and PE groups (the correlation is particularly strong in PE patients; R = −0.9; *p* = 0.009). A low neonatal birth weight is considered to be a negative prognostic factor, increasing the risk of certain neonatal period complications on the one hand and chronic diseases such as diabetes mellitus, hypertension and renal conditions in adult life on the other [15,60]. Thus, the sFlt-1/PlGF ratio seems to be a good predictor of perinatal outcomes dependent on BW in both FGR and/or PE pregnancies.

Kwiatkowski et al. described an inverse correlation of the sFlt-1/PlGF ratio with birth weight of newborns too, and in another study they found significantly lower birth weight in infants of mothers from the group with a high sFlt-1/PlGF ratio compared to groups with a lower ratio in a large group of women with different forms of placental insufficiency [61]. Gaccioli et al. indicated that together with ultrasound examination (biometric measurements plus vascular flow), the sFlt-1/PlGF ratio seems to be a useful additional criterion for the diagnosis of FGR and confirmed that a high sFlt-1/PlGF ratio in fetuses with suspected SGA identifies women at increased risk of adverse perinatal outcomes [29]. PlGF alone had a similar predictive value for these outcomes.

Our results seem to suggest that the coexistence of two entities, FGR and PE, is extremely strongly associated with an elevated sFlt-1/PlGF ratio, which is consistent with observations obtained by Gacciolli et al. [27]. Imaging of the placenta together with Doppler examination of the uterine arteries in combination with markers of angiogenesis, e.g., PlGF may play a role not only in diagnosing FGR but also in predicting adverse perinatal outcomes [29,62,63]. A retrospective cohort study of 274 women with suspected preeclampsia tested the diagnostic potential of PlGF to detect FGR and stillbirths in this group: all six stillbirths had features of FGR, five of which had PlGF levels below the fifth percentile [29,62,63].

Also, studies conducted by Sovio and Visan show that adding the value of the sFlt-1/PlGF ratio to maternal risk factors and ultrasound measurements significantly increases the possibility of early diagnosis of fetuses with FGR and worse perinatal outcomes [30,64].

In the iFGR group, we showed a moderately positive correlation between PlGF and the estimated fetal weight (EFW and EFW expressed in percentiles for gestational age, R = 0.6 and R = 0.73 respectively) and birth weight (BW pc; R = 0.55), as well as with the children’s Apgar scores at 1 and 5 min of life (R = 0.73 and R = 0.71; *p* = 0.005). The same correlations for PlGF were found in perinatal results in the FGR + PE and PE groups. Vrachnis, Wu and Kwiatkowski also described a positive correlation between PlGF and the birth weight of newborns with intrauterine growth restriction [41,65,66]. This proves the great potential of using PlGF not only in PE but also in iFGR and the possibility of using it in planning supervision of the fetus, predicting risks and choosing the moment of delivery in pregnancies complicated by FGR.

The aforementioned publication by Chaiworapongsa et al. focused on measurements of sFlt-1 and PlGF at the time of diagnosis [53]. Later studies showed that high dynamics of sFlt-1 growth in both PE and FGR contribute to its much higher concentrations at the end of pregnancy [67,68]. A study by Herraiz et al., which compared the rate of increase in pregnancies with PE with FGR and those with isolated early-onset FGR, confirmed an increase of 24% per day in the last week before delivery in the group of patients with coexisting early FGR and PE [60]. Andrikos et al., who also focused on early-onset disease, noted an average daily increase in the rate of about 6% in the cases of isolated FGR, and in the group of FGR and concomitant PE, the increase was higher—at the level of 14% per day [28]. In the study of Andrikos, the mean prepartum sFlt-1/PlGF ratio was even slightly, but not statistically significant, higher in the group with isolated FGR compared to the FGR group with concomitant PE (599 vs. 552). As suggested by later studies by Chaiworapongsa et al., the increased angiogenic profile in patients with children diagnosed with SGA was expressed in multiples of the median (MoM), PlGF, sEng, sFlt-1 and their indices, PIGF/sEng and PIGF/sVEGFR-1 and is helpful in identifying those patients who will subsequently develop PE or require delivery less than 34 weeks [69]. Each of these parameters provided significant information about the risk of PE and the possible necessity of induction of labor beyond the data that revealed clinical factors and/or Doppler parameters.

Spanish authors, who carried out serial measurements of the tested substances, emphasize the importance of a rapid increase of the ratio directly before the clinical deterioration and—as a result—delivery in patients with PE, which in clinical practice could be an additional value in choosing the right moment to administer corticosteroids or magnesium sulfate [67,70]. In the last week of pregnancy, the FGR + PE group had a daily increase of 24%, confirming previous reports of a daily increase of 23% in another study in a smaller cohort of patients with early PE [67,70]. According to Chang et al., the mean time from crossing the sFlt-1/PlGF threshold above 85 to delivery is 4.5 weeks in patients with FGR or PE [71]. Schoofs et al. also concluded that patients with FGR have a significant increase in the sFlt-1/PlGF ratio up to 8 weeks before delivery [72].

Our present analysis is consistent with his statement that in the last stage of pregnancy, the sFlt-1/PlGF ratio in iFGR is as high as that in preeclampsia, because the values of the ratio in the two groups were not statistically different. In the Andrikos study, the mean prepartum sFlt-1/PlGF ratio was even slightly, but not statistically significant, higher in the group with isolated FGR compared to the FGR group with concomitant PE (599 vs. 552) [28].

## 5. Conclusions

A positive relationship between PlGF and EFW supports the hypothesis that PlGF might be a helpful tool in FGR pregnancy identification. Furthermore, the highest values of sFlt-1 and the low PlGF in the FGR + PE group seem to suggest accumulation of angiogenic imbalance in preeclamptic pregnancies complicated by FGR, resulting in the strongest alterations in Doppler ultrasound examinations in UA and UtA, and a lower gestational age at delivery compared to the group with isolated PE or FGR (median gestational age 32 vs. 35 weeks, *p* < 0.05).

Positive correlations of sFlt-1/PlGF ratio and maternal sEng levels with UA PI and UA RI in isolated FGR and PE patients as well as negative correlation of PlGF serum concentration with these indices in the FGR + PE group at 48 h before delivery reflect the severity of placental pathology and may suggest that these found angiogenic substance alterations indicate the risk to the fetus and necessity of immediate childbirth.

The correlation of sFlt-1 level with flow measurements in the MCA and CPR, as well as birth weight in the iFGR group, indicates the potential role of this substance as an additional sought-after tool in the care of pregnancy with FGR. It seems that a high or rapid rise of the sFlt-1 level in pregnancies complicated by fetal growth restriction may indicate fetal risk.

### Limitations

We acknowledge some limitations of our study. First, the relatively small size of each group made it difficult to divide patients into early and late onset. Further studies are necessary to demonstrate whether implementation of investigated substances in clinical management brings positive impact, resulting in improvement of prognosis and perinatal outcomes.

## Figures and Tables

**Table 1:** Consensus-based definitions for early and late fetal growth restriction (FGR) [2].

Early FGR: GA < 32 weeks, in absence of congenital anomalies	Late FGR: GA ≥ 32 weeks, in absence of congenital anomalies
AC/EFW < 3rd centile or UA-AEDF Or 1. AC/EFW < 10th centile combined with 2. UtA-PI > 95th centile and/or 3. UA-PI >95th centile	AC/EFW < 3rd centile Or at least two out of three of the following 1. AC/EFW < 10th centile 2. AC/EFW crossing centiles > 2 quartiles on growth centiles 3. CPR < 5th centile or UA-PI > 95th centile

AC, fetal abdominal circumference; AEDF, absent end-diastolic flow; CPR, cerebroplacental ratio; EFW, estimated fetal weight; GA, gestational age; PI, pulsatility index; UA, umbilical artery; UtA, uterine artery.

**Table 2:** Preeclampsia diagnostic criteria established by International Society for the Study of Hypertension in Pregnancy (ISSHP) in 2018 [33].

Preeclampsia
Preeclampsia is gestational hypertension accompanied by ≥1 of the following new-onset conditions at or after 20 weeks’ gestation:
Proteinuria
Other maternal organ dysfunction, including:
AKI (creatinine ≥ 90 umol/L; 1 mg/dL)
Liver involvement (elevated transaminases, e.g., alanine aminotransferase or aspartate aminotransferase > 40 IU/L) with or without right upper quadrant or epigastric abdominal pain
Neurological complications (examples include eclampsia, altered mental status, blindness, stroke, clonus, severe headaches, and persistent visual scotomata)
Hematological complications (thrombocytopenia–platelet count <150,000/μL, disseminated intravascular coagulation, hemolysis)
Uteroplacental dysfunction (such as fetal growth restriction, abnormal umbilical artery [UA] Doppler wave form analysis, or stillbirth)

**Table 3:** Clinical results at time of blood sampling for biochemical tests and ultrasound fetal measurements and Doppler studies.

	FGR + PE		iPE		iFGR		Control	
	I		II		III		IV	
Parameter	Median	Q1–Q3	Median	Q1–Q3	Median	Q1–Q3	Median	Q1–Q3
Gravidity	1	1–2	1	1–2	2	2–3	2	1.5–3
Parity	1	1–2	1	1–2	2	1–2	2	1–2
Gestation (weeks) at sampling	32	28–34	35	33–37	35	33–37	34	31–37
Age (years)	29	27–35	30	27–34	33	30–37	29	28–37
Height (cm)	167	160–170	164	160–168	167	164–171	165	164–168
Weight (kg)	72	66–89	80	72–92	70	67–79	78	68–89
SBP max	170	156–178	156	150–165	127	115–134	114	104–122
DBP max	104	102–111	98	95–105	81.5	76–84	66	62–75
MAP	128	121–131	117.3	114–127	95.7	92–98.7	84	76–89
Proteinuria (mg/24 h)	1438	547–3483	668	295–1981	170	138–192	0	0
Total protein (g/dL)	6.05	5.8–6.3	5.8	5.6–6.3	6.35	6–6.7	6.0	5.5–6.2
Fibrinogen (g/L)	4.4	3.7–5	5.2	4.3–5.6	4.7	4–5.2	3.85	3.5–4.2
INR	0.9	0.86–0.93	0.9	0.9–0.98	0.92	0.9–0.97	1.0	1–1
PT Index(%)	109	105–114	106	100–110	105.3	102–110	98	96–100
PT (s)	10	10.4–9.6	10.3	10.9–9.9	10.4	10.7–9.9	11.1	1.1–10.9
APTT (s)	28.8	26.6–29.6	26.5	25.6–27.3	27.4	26.2–29.6	26.9	25.9–29.4
D-dimers (ng/mL)	1277	1050–1800	1362	1140–1731	1306	951–1670	1359	923–2389
WBC (×10^9^/L)	10.3	8.8–11.4	9.8	9–12.4	9.4	7.5–11.4	9.05	8.4–10.9
RBC (×10^12^/L)	4.16	3.95–4.34	4.0	3.8–4.3	4.2	4–4.3	4.0	3.7–4.2
Hb	12.5	11.8–13.5	12.2	11.4–13.2	12.6	12–13.1	12.25	11.5–12.7
HCT (%)	37	34.3–38.8	34.9	33.7–38.5	36.8	35.6–37.9	35.3	33.9–37.2
PLT (×10^9^/L)	192	147–220	189	144–213	207	178–253	220	179–263
ALT (U/L)	28.5	21–76	24.5	18–46	16	14–28	17	12–18
AST (U/L)	42	30–69	30	25–41	23	20–28	20	15–21
Creatinine (mg/dL)	0.7	0.6–0.8	0.7	0.6–0.8	0.65	0.5–0.7	0.5	0.5–0.6
UA (mg/dL)	7.2	6.7–8.2	6.5	5.5–7.2	5.5	3.95–6.6	4.2	3.2–4.6
Urea	31	26.3–42.1	24.5	20–35	19	19–26	15.6	13.7–17

Q1–Q3—interquartile range; BMI—body mass index; MAP—mean arterial pressure; DBP—diastolic blood pressure; SBP- systolic blood pressure; DBP max—maximum value of diastolic blood pressure; SBP max—maximum value of systolic blood pressure; INR—international normalized ratio; PT—prothrombin time; APTT—activated partial thromboplastin time; WBC—white blood cell count; RBC—red blood cell count; Hb—hemoglobin concentration; HCT—hematocrit; PLT—platelet count; ALT—alanine transaminase; AST—aspartate transaminase; UA—uric acid. Groups of studied women: FGR + PE—women with preeclamptic pregnancy complicated by fetal growth restriction; iPE—women with preeclampsia and appropriate intrauterine fetal growth; iFGR—women with pregnancy complicated by isolated fetal growth restriction; Control group—healthy women with normotensive pregnancies and normal fetal growth; FGR—fetal growth restriction; PE—preeclampsia.

**Table 4:** Characteristics and statistical analysis of ultrasound including Doppler parameters of the study groups (based on results of Kruskal-Wallis test and analysis of variance ANOVA with post hoc RIR Tukey test).

Groups of Studied Women	FGR + PE		iPE		iFGR		Control			
ULTRASOUND MEASUREMENTS INCLUDING DOPPLER FLOW PARAMETERS										
	I		II		III		IV			
Parameter	Median	Q1–Q3	Median	Q1–Q3	Median	Q1–Q3	Median	Q1–Q3	*p* value	Differences
UtPI mean	1.6	1.4–2.0	1.0	1–1	1.6	1.3–2	0.7	0.65–0.75		I, III > IV
Ut PI percentile	100	99–100	91	85–96	100	100–100	52	42–62		I, III > IV
UA PI	1.3	1–2.42	1.0	0.9–1	1.2	1–1.7	0.8	0.65–0.87		I, II, III > IV
UA PI percentile	84	53–100	73	59–82	96	70–100	11	3–46		I, II, III > IV
UA RI	0.8	0.6–1.0	0.6	0.6–0.7	0.7	0.6–0.8	0.6	0.49–0.58		I, III > IV
MCA	1.2	1.1–1.7	1.6	1.5–2	1.3	1.3–1.5	1.6	1.2–1.7	NS	-
MCA percentile	1	1–23	32	12–46	3	1–15	21	8–35	NS	-
CPR	1.2	0.6–1.6	1.6	1.5–1.8	1	0.7–1.6	2.0	1.7–2.2		I, III < IV
CPR pc	1	1–16	19	6–35	1	1–13	51	30–83		I, III < IV
USG—AFI	6.5	3–10	10	8–14	10	7.5–11	11	9–14	NS	-
USG—EFW	1326	708–1714	2760	2167–3173	1915	1464–2255	2607	1773–3351		I < IV
EFWpercentile	1	1–2	56	33–83	2	1–5	64	43–87		I, III < IV
AC	242	217–260	323	299–336	281	245–286	304	260–342		I < IV
AC percentile	1	1–5	58	48–74	4	1–7	57	40–81		I, III < IV
**PERINATAL OUTCOMES**										
Gestational age at birth (weeks)	32	28–34	35	33–37	35	33–37	38	37–39	<0.00005	I, II, III < IV
Birth weight (g)	1370	680–1700	2500	1980–2980	1985	1480–2320	3340	3170–3520	<0.00005	I, II, III < IV
Birth weight percentile	1	1–1	46	22–86	3	1–7	70	47–87	<0.00005	I, III < IV
Apgar 1 min	7	6–8	8	7–10	8	7–10	10	9–10	<0.0005	I, II < IV
Apgar 5 min	7.5	6–9	9	8–10	8	8–10	10	9.5–10	<0.00005	I < IV

*p*-value—statistically significant differences between the groups of studied patients; Q1–Q3—interquartile range; NS—nonsignificant differences between controls and investigated groups. Ut PI mean—mean uterine artery pulsatility index; Ut PI percentile—centile of uterine artery pulsatility index; UA PI—umbilical artery pulsatility index; UA PI percentile—centile of umbilical artery pulsatility index; UA RI—umbilical artery resistance index; MCA PI—middle cerebral artery pulsatility index; CPR—cerebroplacental ratio; CPR percentile—centile of cerebroplacental ratio; AFI—amniotic fluid index; EFW—estimated fetal weight; EFW percentile—centile of estimated fetal weight; AC—abdominal circumference; AC percentile—centile of abdominal circumference. Groups of studied women: FGR + PE—women with preeclamptic pregnancy complicated by fetal growth restriction; iPE—women with preeclampsia and appropriate intrauterine fetal growth; iFGR—women with pregnancy complicated by isolated fetal growth restriction; Control group—healthy women with normotensive pregnancies and normal fetal growth; FGR—fetal growth restriction; PE—preeclampsia.

**Table 5:** Distributions of the values of sFlt-1, sEng, PlGF and the sFlt-1/PlGF ratios in women with isolated PE, isolated FGR, combined PE and FGR and in the control group (based on the results of the Kruskal-Wallis test with Bonferroni adjustment).

	Group	Mean	Median	Q1	Q3	SD	*p* Value
sEng [ng/mL]	(I) FGR + PE	11.9	12.1	11.8	12.2	1.0	
	(II) iPE	10.5	11.5	10.2	11.9	2.4	*p* < 0.001
	(III) iFGR	9.9	11.7	9.2	11.9	3.0	I > IV, II > IV, III > IV
	(IV) Control	6.4	5.8	4.1	8.3	2.9	
PIGF [pg/mL]	(I) FGR + PE	72	42	22	113	62	
	(II) iPE	149	142	27	227	118	*p* < 0.001
	(III) iFGR	216	154	117	221	261	I < IV, II < IV, III < IV
	(IV) Control	851	769	444	1248	480	
sFlt-1 [pg/mL]	(I) FGR + PE	129,263	115,702	14,981	221,278	123,234	
	(II) iPE	87,234	76,345	8614	133,888	99,327	*p* = 0.002
	(III) iFGR	51,193	33,590	13,871	66,994	49,647	I > IV, II > IV
	(IV) Control	9787	8878	5574	10,809	6416	
RATIO sFlt-1/PlGF	(I) FGR + PE	2577	1072	250	2833	4638	
	(II) iPE	1181	314	143	547	3567	*p* < 0.001
	(III) iFGR	408	219	81	846	438	I > IV, II > IV, III > IV
	(IV) Control	18	10	5	24	17	

PlGF—placental growth factor; sEng—soluble endoglin; sFlt-1—soluble fms-like tyrosine kinase-1; SD—standard deviation; Q1–Q3—interquartile range. Groups of studied women: FGR + PE—women with preeclamptic pregnancy complicated by fetal growth restriction; iPE—women with preeclampsia and appropriate intrauterine fetal growth; iFGR—women with pregnancy complicated by isolated fetal growth restriction; Control group—healthy women with normotensive pregnancies and normal fetal growth; FGR—fetal growth restriction; PE—preeclampsia.

**Table 6:** An analysis of correlations between studied substances and ultrasound measurements, including Doppler flow parameters.

Group of Studied Pregnant Women	Ultrasound Parameter	Biochemical Marker	R	*p* Value
CONTROL	EFW	sFlt-1	0.64	0.007
	EFW	sFlt-1/PlGF ratio	0.61	0.01
	AC	sFlt-1	0.53	0.04
	AC	sFlt-1/PlGF ratio	0.54	0.04
	UA PI	PlGF	0.49	0.04
	UA PI	sEng	−0.6	0.008
	UA RI	PlGF	0.6	0.009
	UA RI	sEng	−0.64	0.004
	UA RI	sFlt-1/PlGF ratio	−0.49	0.04
	MCA PI	PlGF	0.75	0.002
	MCA PI percentile	PlGF	0.64	0.01
	MCA PI	sEng	−0.65	0.02
	MCA PI	sFlt-1/PlGF ratio	−0.57	0.03
FGR + PE	UtA PI	PlGF	−0.71	0.01
	UtA PI percentile	PlGF	−0.68	0.02
	UA PI	PlGF	−0.49	0.02
	UA PI percentile	PlGF	−0.52	0.02
	UA RI	PlGF	−0.49	0.02
	CPR	PlGF	0.5	0.02
	CPR percentile	PlGF	0.56	0.009
	EFW	PlGF	0.5	0.02
	EFW percentile	PlGF	0.5	0.02
	AC	PlGF	0.6	0.009
PE(iPE and FGR + PE)	UtA PI	PlGF	−0.55	0.04
	UA PI	sEng	0.33	0.03
	UA PI	PlGF	−0.46	0.003
	UA PI percentile	PlGF	−0.34	0.04
	UA RI	PlGF	−0.5	0.001
	MCA PI	PlGF	0.42	0.01
	MCA PI percentile	PlGF	0.48	0.003
	CPR	PlGF	0.6	0.0001
	CPR percentile	PlGF	0.61	0.0001
	EFW	PlGF	0.43	0.005
	EFW	sEng	−0.35	0.03
	EFW	sFlt-1/PlGF ratio	−0.33	0.03
	AC	PlGF	0.48	0.005
iFGR	UtA PI	sFlt-1/PlGF ratio	0.9	0.04
	UA RI	sFlt-1/PlGF ratio	0.57	0.05
	MCA PI percentile	sFlt-1	−0.57	0.04
	CPR percentile	sFlt-1	−0.66	0.01
	EFW	PlGF	0.6	0.02
	EFW percentile	PlGF	0.73	0.003
	EFW percentile	sEng	−0.58	0.03

*p*-value—statistically significant correlations; R—the Spearman correlation coefficient; Ut PI mean—mean uterine artery pulsatility index; Ut PI percentile—centile of uterine artery pulsatility index; UA PI—umbilical artery pulsatility index; UA PI percentile—centile of umbilical artery pulsatility index; UA RI—umbilical artery resistance index; MCA PI—middle cerebral artery pulsatility index; CPR—cerebroplacental ratio; CPR percentile—centile of cerebroplacental ratio; AFI—amniotic fluid index; EFW—estimated fetal weight; EFW percentile—centile of estimated fetal weight; AC—abdominal circumference; AC percentile—centile of abdominal circumference; PlGF—placental growth factor; sEng—soluble endoglin; sFlt-1—soluble fms-like tyrosine kinase-1. Groups of studied pregnant women: FGR + PE—women with preeclamptic pregnancy complicated by fetal growth restriction; iPE—women with preeclampsia and appropriate intrauterine fetal growth; iFGR—women with pregnancy complicated by isolated fetal growth restriction; Control group—healthy women with normotensive pregnancies and normal fetal growth; FGR—fetal growth restriction; PE—preeclampsia.

**Table 7:** An analysis of correlations between studied substances and perinatal outcomes found in analyzed groups.

Group	Perinatal Outcome	Biochemical Marker	R	*p* Value
PE(iPE and FGR + PE)	Birth weight	PlGF	0.44	0.003
	Birth weight	sFlt-1/PlGF ratio	−0.38	0.01
	Birth weight	sEng	−0.4	0.006
	Birth weight percentile	sFlt-1/PlGF ratio	−0.9	0.009
	Birth weight percentile	sEng	−0.47	0.001
	Apgar 5 min	PlGF	0.35	0.02
FGR + PE	Birth weight	PlGF	0.55	0.008
iFGR	Birth weight percentile	PlGF	0.55	0.04
	Birth weight percentile	sFlt-1	−0.49	0.05
	Birth weight percentile	sFlt-1/PlGF ratio	−0.62	0.02
	Birth weight percentile	sEng	−0.58	0.03
	Apgar 1 min	PlGF	0.73	0.005
	Apgar 5 min	PlGF	0.71	0.005

R—Spearman’s Rank Correlation Coefficient; *p* value—statistical significance; PlGF—placental growth factor; sEng—soluble endoglin; sFlt-1—soluble fms-like tyrosine kinase-1. Groups of studied women: FGR + PE—women with preeclamptic pregnancy complicated by fetal growth restriction; iPE—women with preeclampsia and appropriate intrauterine fetal growth; iFGR—women with pregnancy complicated by isolated fetal growth restriction; Control group—healthy women with normotensive pregnancies and normal fetal growth; FGR—fetal growth restriction; PE—all preeclamptic women (groups iPE and FGR + PE); Birth weight percentile—birth weight percentile according to Akolekar 2018.

**Table 8:** An analysis of correlations between studied substances and biochemical and biophysical and laboratory parameters.

Group	Parameter	Biochemical Marker	R	*p*
iPE	APTT	PlGF	0.44	0.05
	INR	sFlt-1	−0.54	0.01
	PT		0.57	0.007
	INR	sFlt-1/PlGF ratio	−0.53	0.01
	PT		0.52	0.02
	INR	sEng	−0.51	0.03
	PT		0.5	0.03
PE together (iPE and FGR + PE)	INR	sFlt-1	−0.37	0.02
	PT		0.42	0.006
	INR	sFlt-1/PlGF ratio	−0.47	0.001
	PT		0.46	0.002
	INR	sEng	−0.43	0.004
	PT		0.42	0.005
iFGR	SBP	PlGF	−0.68	0.008
	MAP		−0.8	0.0007
	INR		0.66	0.009
	PT		−0.65	0.01
	PT	sEng	0.59	0.03
	urea		0.62	0.03
	Uric acid		0.68	0.01
Control	SBP	sFlt-1	0.5	0.03
	MAP		0.5	0.03
	SBP	sFlt-1/PlGF ratio	0.52	0.02

*p* value—statistical significance; R—Spearman’s Rank Correlation Coefficient; MAP—mean arterial pressure; SBP—maximum value of systolic blood pressure; INR—international normalized ratio; PT—prothrombin time; APTT—activated partial thromboplastin time; PlGF—placental growth factor; sEng—soluble endoglin; sFlt-1—soluble fms-like tyrosine kinase-1. Groups of studied women: FGR + PE—women with preeclamptic pregnancy complicated by fetal growth restriction; iPE—women with preeclampsia and appropriate intrauterine fetal growth; iFGR—women with pregnancy complicated by isolated fetal growth restriction; Control group—healthy women with normotensive pregnancies and normal fetal growth; FGR—fetal growth restriction; PE—all preeclamptic women (groups iPE and FGR + PE).

## Data Availability

Not applicable.
